# Geolocation Patterns, Wi-Fi Connectivity Rates, and Psychiatric Symptoms Among Urban Homeless Youth: Mixed Methods Study Using Self-report and Smartphone Data

**DOI:** 10.2196/45309

**Published:** 2023-04-18

**Authors:** Yousaf Ilyas, Shahrzad Hassanbeigi Daryani, Dona Kiriella, Paul Pachwicewicz, Randy A Boley, Karen M Reyes, Dale L Smith, Alyson K Zalta, Stephen M Schueller, Niranjan S Karnik, Colleen Stiles-Shields

**Affiliations:** 1 Department of Psychiatry and Behavioral Sciences Rush University Medical Center Chicago, IL United States; 2 Rush Medical College Rush University Chicago, IL United States; 3 School of Medicine City University of New York New York, NY United States; 4 Institute for Juvenile Research Department of Psychiatry, College of Medicine University of Illinois Chicago Chicago, IL United States; 5 Center for Health Equity using Machine Learning and Artificial Intelligence (CHEMA) College of Medicine University of Illinois Chicago Chicago, IL United States; 6 Department of Psychological Science School of Social Ecology University of California Irvine Irvine, CA United States

**Keywords:** mHealth, mobile health, smartphones, geolocation, Wi-Fi, youth experiencing homelessness, mobile phone, homelessness, youth

## Abstract

**Background:**

Despite significant research done on youth experiencing homelessness, few studies have examined movement patterns and digital habits in this population. Examining these digital behaviors may provide useful data to design new digital health intervention models for youth experiencing homelessness. Specifically, passive data collection (data collected without extra steps for a user) may provide insights into lived experience and user needs without putting an additional burden on youth experiencing homelessness to inform digital health intervention design.

**Objective:**

The objective of this study was to explore patterns of mobile phone Wi-Fi usage and GPS location movement among youth experiencing homelessness. Additionally, we further examined the relationship between usage and location as correlated with depression and posttraumatic stress disorder (PTSD) symptoms.

**Methods:**

A total of 35 adolescent and young adult participants were recruited from the general community of youth experiencing homelessness for a mobile intervention study that included installing a sensor data acquisition app (Purple Robot) for up to 6 months. Of these participants, 19 had sufficient passive data to conduct analyses. At baseline, participants completed self-reported measures for depression (Patient Health Questionnaire-9 [PHQ-9]) and PTSD (PTSD Checklist for DSM-5 [PCL-5]). Behavioral features were developed and extracted from phone location and usage data.

**Results:**

Almost all participants (18/19, 95%) used private networks for most of their noncellular connectivity. Greater Wi-Fi usage was associated with a higher PCL-5 score (*P*=.006). Greater location entropy, representing the amount of variability in time spent across identified clusters, was also associated with higher severity in both PCL-5 (*P*=.007) and PHQ-9 (*P*=.045) scores.

**Conclusions:**

Location and Wi-Fi usage both demonstrated associations with PTSD symptoms, while only location was associated with depression symptom severity. While further research needs to be conducted to establish the consistency of these findings, they suggest that the digital patterns of youth experiencing homelessness offer insights that could be used to tailor digital interventions.

## Introduction

Homelessness and housing instability are experiences that impact roughly 4.2 million youths annually [1]. Youth experiencing homelessness face complex challenges that span multiple domains, including social and mental health concerns [2-4]. As such, youth experiencing homelessness have been conceptualized as having socially complex needs, meaning that they have heterogeneous and potentially overlapping experiences (eg, adverse childhood experiences) or marginalized identities (eg, minoritized racial, ethnic, sexual, or gender identity) [5]. Reflecting this lens of socially complex needs, previous research has examined sexual trauma, gang affiliation, familial substance use, and parental abuse as contributing factors to the experience of homelessness in adolescents and young adults (eg, young people under the age of 25 years) [3,4,6]. These experiences, coupled with their lack of stable housing, are associated with a greater likelihood of high-risk behaviors, negative psychosocial sequelae, and trauma symptoms [7]. As such, youth experiencing homelessness are in incredibly high need of mental and behavioral health services, yet are a highly underserved population [2].

A growing literature is focused on the use of digital health interventions (DHIs) to support youth experiencing homelessness [8,9]. Indeed, access issues unique to youth experiencing homelessness, such as transience, highlight the potential benefits of mobile DHI as a care delivery mechanism. Further, access to mobile devices is high for youth experiencing homelessness, with a recent review identifying that 53%-100% of homeless participants across 17 studies owned a mobile phone or smartphone [10]. However, given their socially complex needs [5], youth experiencing homelessness are likely to experience multiple barriers and specific user needs related to mobile DHI. For example, the potentially prohibitive cost of mobile data plans may limit this group’s ability to access mobile interventions to times when they are within the range of publicly accessible Wi-Fi connections. Reliance on publicly accessible spaces may be impacted by multiple factors, including business hours (eg, accessing a cafe’s Wi-Fi when open), personal safety in specific spaces, and public health guidance (eg, social distancing mandates in the context of the COVID-19 pandemic) [11]. Although DHI might be used to support the mental health of youth experiencing homelessness, it is important to recognize that mental health symptoms might create a barrier to its access and use. The mental health symptoms of youth experiencing homelessness may actually impact the consistency or frequency of using resources in publicly accessible spaces (eg, trauma symptoms leading to avoidance of certain places or situations). While multiple recent works cite the need to use human-centered design methodologies to inform the design of DHI specifically to the needs of populations with socially complex needs [12-15], little is known about design specific for youth experiencing homelessness.

Passive data collection involves generating data with little to no active participation from an individual [16]. While there are many forms of passive data collection, Wi-Fi connectivity and movement patterns may be collected without additional work on the part of youth experiencing homelessness. Indeed, mobile devices are capable of tracking Wi-Fi usage patterns, such that the duration and frequency of Wi-Fi connections may be measured, as well as the use of public versus private Wi-Fi networks. Accessible Wi-Fi may be a key factor for youth experiencing homelessness to access DHI and other web-based resources [13,15]. Further, geolocation data collected by mobile devices can indicate the level of variance in an individual’s movement, referred to as entropy [17]. Entropy and broader indicators of movement collected via mobile devices have been found to correlate with or predict depressive mood states in housed populations [18-20]. In this literature, individuals’ homes were believed to be one of the key locations for the correlations found. Passive data collection such as this may evolve to effectively inform just-in-time adaptive intervention (JITAI) methodologies [21], such that DHI could be provided at “the right time” with “the right amount” based on a user’s inner (emotional) and outer (location) context. Given the burdens placed on youth experiencing homelessness, they may be a prime beneficiary of JITAIs, but there are limited data to inform such work. Indeed, as youth experiencing homelessness are, by definition, unstably housed, it is unclear how the current literature extends to this group. Further, the geolocation data collected with housed populations has focused on depression, whereas youth experiencing homelessness are likely to experience both depressive and trauma-related symptoms [22]. It is reasonable to theorize that unhoused populations without a fixed set of locations, like home, might show more diffuse patterns relative to psychiatric symptoms and geolocation. Findings from this line of research could inform DHI for youth experiencing homelessness and adaptations to better fit the socially complex needs of this population.

Given the lack of passive data collection conducted with youth experiencing homelessness combined with the potential for advancing DHI for this unique population, the purpose of this study was to pilot an examination of the Wi-Fi use patterns and geolocation and the associations of these factors with mental health symptoms reported by adolescents and young adults experiencing homelessness in a major city in the United States. Specifically, secondary data analyses were conducted to explore the frequency of connectivity to public or private Wi-Fi networks, GPS data dispersion, and self-reported depression and trauma symptoms for youth experiencing homelessness enrolled in a multicomponent mobile phone–based DHI. Depression and trauma symptoms were selected for these analyses given their high prevalence in this population as well as their impact on the broader mental health outcomes of youth experiencing homelessness [22]. Based on previous findings [23,24], it was hypothesized that youth experiencing homelessness would show: (1) high levels of entropy in terms of location movement, meaning time would be spent relatively uniformly across different locations [17], (2) a reliance primarily on public Wi-Fi for connection, and (3) less time spent on Wi-Fi than the average American (ie, approximately 900 minutes daily) [25].

## Methods

### Participants

As previously reported [26], 35 adolescents and young adults (age 18-24 years) experiencing homelessness were recruited through shelters in Chicago, Illinois, United States, from January 2016 to November 2017. Potential participants were referred by flyers posted in shelters or by their case managers. The 2 main recruitment site shelters run by the same organization were Shelter A, a hybrid shelter that provides emergency housing for up to 30 days and a longer-term residential program that houses youth for several months; and Shelter B, a long-term shelter that supports pregnant and parenting youth. Screenings occurred at the shelters, and informed consent was collected digitally on study tablets. To participate in the study, individuals needed to be aged 18 to 24 years, speak English, and be homeless, as defined by lacking “a fixed, regular, and adequate nighttime residence” [27]. Participants were excluded if they displayed cognitive impairment that would prevent them from giving meaningful consent (as assessed via the shelter case worker), were currently engaged in psychotherapy or legal proceedings that would interfere with study procedures (eg, on probation), were experiencing significant suicidal ideation (as assessed via the Mini International Neuropsychiatric Interview), or displayed behaviors that would preclude participation in the study, such as previous enrollment in the pilot study or an unwillingness to adhere to study procedures.

### Ethics Approval

The study was approved by the institutional review board at Rush University Medical Center (reference number: 14112402). Potential participants deemed eligible for screening completed informed consent and had the opportunity to ask questions. All study data were deidentified to ensure privacy and confidentiality. Further details of the study population and procedures are available [26].

### Procedures

While this study only examined baseline self-report data that were collected prior to intervention (detailed below), the primary data set was part of a multicomponent DHI [26], wherein participants received 1 month of coaching support (ie, phone calls, SMS text messages) concurrently with a smartphone (Android Nexus V) preloaded with 3 apps associated with the intervention and a service and data plan for up to 6 months. Purple Robot (Center for Behavioral Intervention Technologies) [28], one of the apps associated with the intervention, extracted different metadata (eg, Wi-Fi connectivity history, GPS location, and related timestamps) for each participant during the intervention time period of 180 days. Extracted metadata were collected via remote data transmission from the device and maintained on a secure and encrypted server maintained by Northwestern University. Participants were made aware of this process and provided consent to share data in this manner. Participants who lost or severely damaged their phones were permitted 1 replacement device.

### Public and Private Wi-Fi Measurement

For this study, a public Wi-Fi network was defined as a network that the general public has access to and is able to connect to, while a private Wi-Fi network was all other types of network. Network names and IP addresses were individually examined to determine whether they were public or private networks. Specifically, if there was any use of “guest” in the network name (eg, “Starbucks-Guest”) or a common name, then the network was classified as public. All other network names were deemed to be private.

### Location Measurement

Participant location data were tracked through Purple Robot [28]. Purple Robot continuously stored the latitude and longitude coordinates of each participant during the intervention period, along with the subsequent Unix timestamp. Unix time is a time representation that is frequently used in computing. Time is given as an integer value, representing the number of seconds that have passed between the current time and January 1, 1970. It allows timestamps to be represented more succinctly while allowing for easy conversion to other forms if needed.

### Measures

Participants completed self-report questionnaires at enrollment and 1 month following the completion of the intervention [26].

#### Demographics

At baseline, participants were asked to provide demographic information on age, gender, race, ethnicity, sexual orientation, enrollment in school, employment, education level, and age of first homelessness experience.

#### Psychiatric Assessments

##### Patient Health Questionnaire-9 (PHQ-9)

The PHQ-9 is a 9-item self-report measure that assesses depressive symptoms [29]. Scores on the PHQ-9 range from 0 to 27, with higher scores indicating greater depressive symptoms. Scores 5, 10, 15, and 20 represent the cut points for mild, moderate, moderately severe, and severe depressive symptoms. This was measured following enrollment but prior to the intervention and demonstrated good internal consistency in the current sample (α=.92).

##### PTSD (Posttraumatic Stress Disorder) Checklist for DSM-5 (PCL-5)

The PCL-5 is a 20-item self-report measure that assesses core symptoms of PTSD based on the *Diagnostic and Statistical Manual of Mental Disorders*, Fifth Edition (DSM-5) criteria [30]. Scores on the PCL-5 range from 0 to 80, with a score of 33 or higher suggesting likely PTSD. Scores for key PTSD symptom clusters, including intrusion, avoidance, negative alterations in cognition and mood, and alterations in arousal and reactivity, may also be calculated using the PCL-5. The PCL-5 was also collected prior to the intervention and demonstrated high internal consistency in the current sample (α=.96).

#### Data Analysis

**Figure 1:**
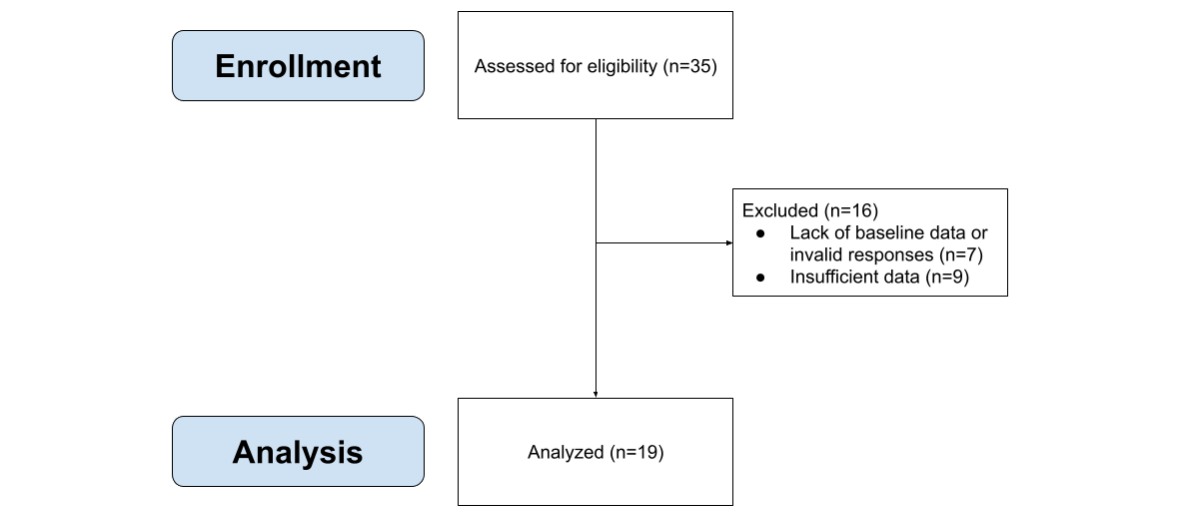
Flow of participants in this study. Participants who had less than 14 days of Wi-Fi data were excluded from the study in an attempt to keep the study representative. Devices that were designated as test phones were also excluded from the sample.

Due to the large size of the collected data (50+ GB), different elements of the data were pulled separately for analysis. A script was written in Python that extracted all of the GPS and Wi-Fi usage data for each participant. The sample began with 35 participants. Participants (n=7) who lacked baseline surveys or provided invalid measures were excluded from the sample, as in our prior report on this data [26]. All participants with less than 14 days of Wi-Fi data were excluded from the analytic sample (n=9) for both GPS and Wi-Fi analyses. To establish a baseline, 2 weeks were selected to establish a baseline, as 14 days may account for any disruptions that may occur on a given weekday or weekend (eg, an out-of-the-ordinary Tuesday would be counterbalanced by a more typical Tuesday the following week). Three subjects lost or had their study smartphone stolen, and they were provided replacement devices. In these cases, the data from the first phone was connected and appended to the new device, and this data was analyzed as a single sequence to align with participants who had a single device for the entire study. Data incidentally collected beyond the study time period of 180 days were excluded, as these data were transmitted for some participants who did not remove the Purple Robot app as instructed. The average number of days of Wi-Fi usage for the participants included in the sample was 87. Our final analytic sample was 19 participants. Figure 1 depicts the flow of participants from the primary intervention study through this study.

#### Wi-Fi Data

The mean Wi-Fi time was calculated in a heuristic mode using the following logic: if the difference between 2 consecutive Unix timestamps was greater than 1000 (approximately 15 minutes), it was assumed that the participant got off the Wi-Fi network for an amount of time and then reconnected. Time spent on Wi-Fi was analyzed as the difference between the previous Wi-Fi time and the next Wi-Fi time. A running total of these times were then generated for each participant. In addition, we examined some of the most popular types of public Wi-Fi networks that were accessed, such as hotels, libraries, universities, and so on, and the rate at which participants connected to them. We then manually classified each network as either public or private.

#### Location Data

As GPS data were collected multiple times each minute, the data were aggregated into 15-minute time intervals for ease of analysis. After the amalgamation of this data, the density-based spatial clustering of applications with noise (DBSCAN) algorithm was used to cluster the location data for each participant based on proximity [31]. This approach was chosen due to its prior demonstrated utility as well as flexibility in defining intuitively meaningful distance and the number of points per cluster for the algorithm. The DBSCAN algorithms were set with a maximum within-cluster distance of 0.5 km and a minimum of 5 points per cluster. We then determined the number of clusters for each individual. Entropy was computed following Saeb et al [17], which represents the variability of time a participant spent across identified clusters. Higher entropy values indicate that the participant distributed time more uniformly across clusters. Normalized entropy was also examined. This metric has the same general meaning as entropy but is not affected by the number of clusters. Since the number of clusters differs by individual, we sought to ensure the results were robust to either characterization of entropy.

#### Mental Health Data

In examining the relationship between location (represented through entropy and normalized entropy) or Wi-Fi usage and mental health measures, models were adjusted for whether participants reported employment or school. These factors were judged to impact both the ability to access Wi-Fi (through free access to school or work routers) and movement (through being based in a single location for an extended period of the day or week). Entropy, normalized entropy, and Wi-Fi usage were included as predictors of both PTSD severity (PCL-5) and depression severity (PHQ-9) using separate linear regression analyses. The movement was also analyzed by shelter site, as the shelters had different durations of stay (Shelter A being shorter-term, and for emergency housing, Shelter B being longer-term by comparison). Covariates were otherwise limited due to power and sample size concerns. The effect size using *R*^2^, representing the proportion of variability in psychiatric outcomes that can be explained using entropy or Wi-Fi usage, was emphasized due to the small sample. Entropy estimation using DBSCAN and statistical analyses predicting mental health outcomes were conducted in Python (version 3.10.4; Python Software Foundation).

## Results

### Participants

A total of 19 participants were included in this study. Table 1 displays the participant characteristics. The average age of participants was 19 years, with 17 years being the average age of first experiencing homelessness. Most of the current sample identified as cisgender female (68.4%), non-Hispanic or Latinx (73.7%), Black or African American (68.4%), heterosexual or straight (78.9%), not enrolled in school (52.6%), or employed (78.9%). There was no evidence to suggest differences in demographic variables (ie, age, gender, ethnicity, race, sexual orientation, student status, or employment status) between the current sample and the larger intervention sample (*P*s>.30) [26]. At the time of recruitment for the study, the youth had been staying in the shelter for at least 4 nights.

**Table 1:** Participant demographics^a^.

Characteristics		Values
Age (years) (n=16), mean (SD; range)		19.00 (0.89; 18-20)
Age of first homelessness experience (years) (n=18), mean (SD; range),		17.39 (1.42; 14-20)
**Gender, n (%)**		
	Cisgender female	11 (57.9)
	Cisgender male	7 (36.8)
	Transgender female	1 (5.3)
**Ethnicity, n** **(%)**		
	Hispanic or Latinx	14 (26.3)
	Non-Hispanic or Latinx	5 (73.7)
**Race, n** **(%)**		
	Black or African American	11 (57.8)
	Multiracial	4 (21.1)
	White	3 (15.8)
	Unknown	1 (5.3)
**Sexual orientation, n** **(%)**		
	Heterosexual or straight	14 (73.7)
	Gay or lesbian	2 (10.5)
	Bisexual or pansexual	2 (10.5)
	Don’t know	1 (5.3)
**Enrolled in school, n** **(%)**		
	Yes	11 (57.9)
	No	8 (42.1)
**Employed, n** **(%)**		
	Yes	3 (15.8)
	No	16 (84.2)
**Highest level of education, n (%)**		
	Less than high school diploma	7 (36.8)
	Regular high school diploma	6 (31.6)
	GED^b^ or alternative degree	2 (10.5)
	Vocational or training school	1 (5.3)
	Some college or associate’s degree	3 (15.8)

^a^One participant identified their race as “Middle Eastern” but was classified as White based on current census protocols; however, their ethnicity as Middle Eastern and North African is noted here.

^b^GED: General Educational Development test.

### Wi-Fi Use

**Figure 2:**
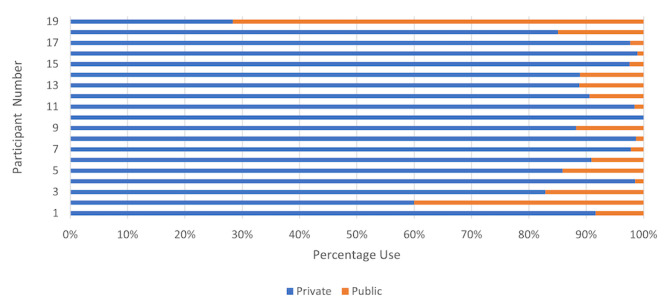
Participant distribution of private and public Wi-Fi network usage.

The mean Wi-Fi use for youth experiencing homelessness varied greatly, ranging from 1 minute to 628 minutes per day. Even by conservative estimates, the vast majority of participants spent less time on Wi-Fi than the average American adult (ie, approximately 900 minutes daily) [25]. Youth experiencing homelessness that had a large average Wi-Fi time typically relied on 1 Wi-Fi network for the majority of their connections, suggesting that they had reliable access to a private network. Most participants relied on private Wi-Fi usage more than public Wi-Fi, with 18 participants (95%) having greater than 50% of their total Wi-Fi usage on a private network. Figure 2 shows the distribution of each participant.

### Patterns in Movement

Three participants briefly traveled out of state, and these data points were not used for cluster generation. The mean number of clusters among participants was 7.37 (SD 4.94; range 1-19). The cluster generation revealed distinct patterns of movement, with some movements corresponding to bus and train lines. The clusters also indicated that the daily distance covered ranged from 1 mile to over 80 miles.

### Associations With Mental Health Symptoms

#### Wi-Fi Usage

After adjusting for whether participants were students or employed, the average number of Wi-Fi usage minutes was a significant predictor of PCL-5 scores (*b*=.09, *P*=.006). Higher levels of Wi-Fi usage were associated with higher PTSD symptom severity scores. Further, Wi-Fi accounted for approximately 15% of the variance in PCL-5 (*R*^2^=.15). Evidence did not suggest a significant relationship between Wi-Fi usage minutes and depressive symptoms (*P*=.500, *R*^2^<.01). The low proportion of the variance in PHQ-9 accounted for by Wi-Fi further suggests that this nonsignificant association is unlikely to be due to low statistical power.

#### Patterns in Movement

Significant relationships also existed between entropy and both PCL-5 (*b*=–30.55, *P*=.007) and PHQ-9 scores (*b*=–7.08, *P*=.045; see Table 2), suggesting that higher entropy values were associated with lower PTSD severity scores and depression. This significant association was also apparent using normalized entropy (ie, the entropy that is normalized by the number of location clusters; PCL-5 *b*=–58.13, *P*=.02; PHQ-9 *b*=–17.69, *P*=.02). However, this relationship was only seen in the Shelter A site for the PCL-5 (*P*=.006), not the Shelter B site (*P*=.16; see Table 3). We chose to further examine these findings by a shelter because of the longer-term nature of Shelter B as compared to Shelter A, which generally had shorter-term stays for emergency housing.

**Table 2:** Relationships between mental health symptoms and location metrics.

	Entropy			Normalized entropy			Number of clusters		
	*b*	*P* value	*R* ^2a^	*b*	*P* value	*R* ^2^	*b*	*P* value	*R* ^2^
PCL-5^b^	–30.55	.007	0.30	–58.13	.022	0.23	1.33	.30	0.12
PHQ-9^c^	–7.08	.045	0.15	–17.69	.019	0.21	0.39	.34	0.06

^a^Parameter estimates represent multivariable regression values, and all models controlled for shelter and whether the participant was employed or in school. However, *R*^2^ values represent the strength of bivariate relationships between location and mental health variables without including the contributions of these covariates.

^b^PCL-5: PTSD Checklist for DSM-5.

^c^PHQ-9: Patient Health Questionnaire-9.

**Table 3:** Relationship between mental health symptoms and entropy by shelter.

	Shelter A			Shelter B		
	*b*	*P* value	*R* ^2a^	*b*	*P* value	*R* ^2^
PCL-5^b^	–45.21	.006	0.54	–24.31	.16	0.26
PHQ-9^c^	–8.75	.19	0.19	–5.14	.18	0.24

^a^Parameter estimates represent multivariable regression values, and all models controlled for whether the participant was employed or in school. However, *R*^2^ values represent the strength of bivariate relationships between location and mental health variables without including the contributions of these covariates.

^b^PCL-5: PTSD Checklist for DSM-5.

^c^PHQ-9: Patient Health Questionnaire-9.

## Discussion

This study examined the Wi-Fi tendencies, movement patterns, and associations of these behaviors with mental health symptoms reported by a research sample of youth experiencing homelessness. First, we found a relationship between trauma and depressive symptoms and the degree to which individuals follow geographic patterns. Participants who spent time more uniformly distributed across clusters had lower average trauma and depression severity. Further, for trauma-related symptoms, differences emerged for entropy based on the shelter site. Second, despite varied Wi-Fi use, the majority of participants demonstrated less overall Wi-Fi connectivity than the average American adult (ie, 900 minutes) [25]. Finally, greater time on Wi-Fi networks was associated with higher trauma-related symptom severity, which was represented by a fairly large effect size. Contrary to our original hypotheses, the majority of participants relied more heavily on private connections than public ones.

Youth experiencing homelessness with more self-reported trauma symptoms also had higher use of Wi-Fi. Indeed, each additional minute of average Wi-Fi use predicted a rise in the PCL-5 total score by nearly a point. Given the small sample size and the observational nature of these data, we are cautious to overinterpret the findings. However, this association between increased use and trauma symptoms may reflect how Wi-Fi may support the coping techniques used by youth experiencing homelessness. Indeed, youth have endorsed technology use as a means to distract themselves from distress, seek social support, or as an activity when having difficulty sleeping [32]. Of note, while we hypothesize that this usage was driven by a coping strategy, the efficacy of this strategy in the long term may be small (ie, trauma symptoms may not be impacted through the use of this strategy). Our study also had an intervention element during the first month, and this may have factored into the degree to which youth experiencing homelessness sought Wi-Fi connectivity, especially for those with psychiatric symptoms. We did not, however, find a significant association between depressive symptoms and Wi-Fi usage.

Time spent across spaces was not evenly spread, particularly for youth experiencing homelessness with higher mental health symptoms. Depressive symptoms have previously been associated with or predicted by patterns of mobility, suggesting that greater depressive symptoms lead participants to “hunker down” in identified safe places [18-20]. Further, this behavioral pattern was anticipated for youth experiencing homelessness with trauma histories, as such individuals are more likely to attempt to create more structure for themselves in routine spaces [33]. However, in this study, youth experiencing homelessness with greater mental health symptoms tended to have a less uniform distribution of time across different clusters and therefore tended to be more concentrated in fewer clusters. This finding aligns with the “hunker down” pattern mentioned above. While shelter site differences were noted for those with trauma symptoms, these differences may be attributed to multiple factors. For example, youth experiencing homelessness at Shelter B were more likely to have prenatal or childcare responsibilities, which might have impacted their movement patterns. However, despite many possible mitigating factors, further examining this issue is beyond the scope of this study.

The advancement of mobile sensing technologies may promote a better understanding of the “right” time, place, and context for DHI to intervene with youth experiencing homelessness [34]. First, the relationship between mental health symptoms, Wi-Fi use, and entropy may be a potential avenue to support DHI that uses JITAI methodologies [21]. Indeed, the current findings add to a growing body of literature suggesting that movement patterns [18-20] and increased Wi-Fi use [35-37] may indicate when youth experiencing homelessness are at increased risk for experiencing mental health symptoms. Further, the limited access to and use of Wi-Fi by youth experiencing homelessness compared to the general population highlights the need to consider how much and when DHI requires Wi-Fi or data use [38,39]. Additionally, the disparity in Wi-Fi use among the current population of youth experiencing homelessness compared with the broader US population is a further reminder of inequitable access to a commonly used tool for multiple domains of current life, not just mental health. These issues, combined with the socially complex needs of youth experiencing homelessness, may make tailored DHI approaches, such as JITAI, more likely to fit the needs of youth experiencing homelessness, provided ongoing needs assessments occur and increases inequitable access to broadband internet continue. Second, several previous studies have demonstrated the efficacy of DHIs in minimizing risky behaviors and assisting with healthy social contacts [8].

To our knowledge, this study is the first of its kind to present the Wi-Fi use and mobility patterns of youth experiencing homelessness in an urban setting. However, specific limitations should be considered while interpreting the findings. Beyond the limitations noted from the primary outcome trial (eg, iterative inclusion criteria (concurrent counseling, substance dependence) [26]), the methodology of this study required many decisions around data inclusion. Without clear guidance from prior research, choosing hyper-parameters for DBSCAN algorithms and which participants to exclude were based on metrics that were deemed to be the best fit for this study. Additionally, some participants experienced lost or stolen phones, thus adding a confounding variable around data inclusion. The small sample size in this study could also limit generalizability; thus, results should be interpreted with attention to this consideration. Another limitation was the heuristic-based approach to classifying public and private Wi-Fi networks, as this method was open to a certain degree of error in categorization when the type of network was unclear. It is also important to note that the data for this study were collected prior to the COVID-19 pandemic. The degree to which the pandemic changed or altered the use of mobile devices is an open question. Future research should continue to recruit and engage youth experiencing homelessness to explore these variables further. Specifically, future research should explore the digital behaviors of this population in different settings (eg, rural) and with larger sample sizes to account for confounding variables that are expected for a population with socially complex needs, such as lost or stolen phones.

We also want to comment directly on the research ethics of the current work. Using mobile sensing technology with youth experiencing homelessness raises concerns regarding digitally monitoring a sensitive population as well as potential challenges that might arise if certain types of information are learned through such monitoring [40]. This study was the result of the considerable partnership and codevelopment with the homeless shelter network and youth experiencing homelessness themselves [40,41]. Additionally, we obtained a certificate of confidentiality to ensure that data would not have to be shared in a manner that might negatively impact the participants. Conducting such work requires building trust with potential participants and community participants, as well as promoting transparency between study documents such as advertising materials, consent forms, and the intervention materials themselves.

This study piloted the assessment of Wi-Fi usage and movement patterns in youth experiencing homelessness and explored associations between these passively sensed data and mental health symptoms. Promisingly, the findings demonstrated that homeless youth access Wi-Fi, often through private networks, but access Wi-Fi less than the general population. Further, their patterns are associated with mental health symptoms, with less movement between clusters and more Wi-Fi being correlated with depression or trauma and trauma, respectively. This study adds to a growing body of literature exploring how DHI might better reach and serve youth experiencing homelessness. Continued assessment and engagement with youth experiencing homelessness can support the tailoring of DHI to better reach and serve the socially complex needs of this population.

## Abbreviations

DBSCAN: density-based spatial clustering of applications with noise
DHI: digital health intervention
JITAI: just-in-time adaptive intervention
PHQ-9: Patient Health Questionnaire-9
PCL-5: PTSD Checklist for DSM-5
PTSD: posttraumatic stress disorder

## Data Availability

The data sets generated during or analyzed during this study are not publicly available as the nature and volume of the data are inappropriate for public posting. Specifically, the data contains geocodes and phone data that would risk disclosure of identity.
